# The β-goblin gene architecture in individuals with and without sickle cell disease in Nigeria: Implications for β-thalassaemia trait diagnosis

**DOI:** 10.4102/ajlm.v15i1.2985

**Published:** 2026-01-28

**Authors:** Oluwatoyin A. Babalola, Biobele J. Brown, Foluke Fasola, Jing Zhang, Yonglan Zheng, Abayomi B. Odetunde, Adeyinka G. Falusi, Olufunmilayo Olopade

**Affiliations:** 1 Institute for Advanced Medical Research and Training, College of Medicine, University of Ibadan, Ibadan, Nigeria; 2 Department of Molecular Biology and Biotechnology, College of Natural and Applied Sciences, Chrisland University, Abeokuta, Nigeria; 3 Department of Paediatrics, College of Medicine, University of Ibadan, Ibadan, Nigeria; 4 Department of Haematology, College of Medicine, University of Ibadan, Ibadan, Nigeria; 5 Section of Hematology/Oncology, Department of Medicine, University of Chicago, Illinois, Chicago, United States

**Keywords:** β-thalassaemia trait, β-globin gene, sickle cell disease, haplotype, variants, Yoruba population, Nigeria

## Abstract

**Background:**

β-thalassaemia is considered rare in Africa; however, recent screening-based studies suggest a β-thalassaemia trait prevalence of 6% – 10% among individuals with sickle cell disease (SCD) and up to 25% in those without SCD. Co-inheritance with SCD may modify disease severity, highlighting the need for molecular confirmation.

**Objective:**

To ascertain the prevalence and genetic basis of β-thalassaemia trait in Nigerians with and without SCD.

**Methods:**

We recruited 260 participants (130 per group; aged 3 years – 69 years, median [interquartile range] = 16 [9–29]). Haemoglobin fractions were analysed using high-performance liquid chromatography, and full blood counts were obtained. A 1.6 kb region of the β-globin gene was amplified and sequenced by Sanger sequencing. Variants were annotated and haplotypes constructed. An additional 26 samples from a separate SCD cohort were also genotyped.

**Results:**

Molecular analysis revealed a β-thalassaemia trait prevalence of < 1% in both groups, contrasting with recent screening-based reports. In addition to sickle cell, haemoglobin C, and β-thalassaemia mutations, eight other variants were identified, three of which were unique to SCD patients and in linkage disequilibrium. Sickle cell and haemoglobin C mutations occurred on the major ancestral haplotype, whereas the only β-thalassaemia mutation detected (rs33915217C>A) was associated with a minor ancestral haplotype atypical of Africa. Two rare variants (rs537944366T>C and rs33915217C>A) are reported for the first time in the Yoruba population.

**Conclusion:**

These findings indicate a low prevalence of β-thalassaemia trait in Nigeria and underscore the need to re-evaluate diagnostic approaches in African populations for optimal clinical management of SCD and other anaemias.

**What this study adds:**

This study provides the first molecular confirmation of the low prevalence of β-thalassaemia trait in the Yoruba population. It identifies two rare variants, including a β-thalassaemia mutation on a minor, atypical haplotype, and highlights the limitations of high-performance liquid chromatography, underscoring the importance of genetic testing for accurate diagnosis.

## Introduction

β-thalassaemia is primarily caused by point mutations and, less commonly, by deletions or insertions in the β-globin gene locus.^1^ This gene is highly heterogeneous, with approximately 954 documented variants, over 300 of which are associated with β-thalassaemia.^2^ β-thalassaemia major arises from the complete absence of β-globin chain production, while β-thalassaemia trait (minor) and β-thalassaemia intermedia involve a reduction in β-globin synthesis. All three forms result in decreased production of adult haemoglobin (HbA),^3,4,5^ leading to anaemia of varying severity, depending on the mutation type and zygosity.^4^

While β-thalassaemia is globally distributed, its prevalence varies. Historically, it has been endemic in the Mediterranean, the Middle East, and Southeast Asia, with the highest carrier frequencies reported in regions such as Cyprus, Sardinia, and parts of Southeast Asia.^6,7^ The global carrier rate is estimated at approximately 1.5%.^5^ However, patterns are shifting as a result of increased migration and interethnic marriages, leading to cases in previously low-prevalence areas.^5,7^

In West Africa, particularly Nigeria, β-thalassaemia was once considered rare. However, recent studies suggest a rising prevalence. In Nigeria, population-based screening studies have estimated a β-thalassaemia trait carrier rate of up to 25%,^8,9,10^ while neighbouring countries such as Liberia and others in sub-Saharan Africa have reported estimation rates ranging from 9% to 20%.^11,12^ These estimates are commonly based on elevated HbA_2_ (> 3.5%) and HbF (> 1%) levels determined by high-performance liquid chromatography (HPLC). In contrast, earlier methods such as starch-block electrophoresis, used by Esan, reported a prevalence of < 1%, using a diagnostic cutoff of HbA_2_ > 3.8%.^13^

Sickle cell disease (SCD), which also results from a point mutation in the β-globin gene, may co-exist with β-thalassaemia. Among Nigerian patients with SCD, HPLC-based screening suggest a prevalence of co-inherited β-thalassaemia trait between 6% and 10%.^14,15^ However, a molecular-based study involving children under five in Northern Nigeria reported a much lower prevalence of 0.4% among children with SCD, and none among those without.^16^ The β-thalassaemia trait (β^+^-thal), when co-inherited with SCD, tends to ameliorate the clinical severity of SCD. Individuals with this compound heterozygous genotype (HbSβ^+^-thal) typically experience a milder disease course and fewer vaso-occlusive crises, similar to the HbSC genotype.^17^ Hence, it is expected to be at a higher prevalence in individuals with SCD compared to the general population, contrary to recent reports.

Diagnosis of β-thalassaemia trait in Nigeria commonly relies on HbA_2_ and HbF thresholds as the first initial screening process, but several confounding factors can elevate HbA_2_ levels, including α-thalassaemia,^16,18^ vitamin B12 or folate deficiency, antiretroviral therapy, and hyperthyroidism.^19^ Notably, α-thalassaemia itself is prevalent in Nigeria, with rates exceeding 35%.^18,20^ Nigeria bears the highest burden of SCD globally, yet the true prevalence and genetic characteristics of β-thalassaemia trait in this population remain uncertain. This study was therefore conducted to investigate the β-globin gene architecture in individuals with and without SCD in Nigeria by integrating molecular sequencing, variant annotation, and haplotype analysis to determine the prevalence and genetic spectrum of β-thalassaemia trait and to assess whether this differs significantly between the two groups. This study is among the first to use molecular genotyping to re-evaluate the prevalence of β-thalassaemia trait in Nigerians.

## Methods

### Ethical considerations

This study was approved by the University of Ibadan, University College Hospital, Ibadan Institutional Ethics Review Committee, College of Medicine, University of Ibadan (reference number: UI/EC/16/0195). Written consent form was obtained from each participant. The design and implementation of the research protocol were in accordance with the principles of The Declaration of Helsinki. A code was assigned to each participant and access to their demographic information stored on the computer was restricted to a few authorised individuals. This manuscript adheres to the Strengthening the Reporting of Observational Studies in Epidemiology guidelines for the reporting of observational studies.

### Study design

This is a cross sectional study of 130 SCD patients in steady state attending University College Hospital Paediatric Outpatient Clinic and Haematology Clinic in Ibadan, Nigeria. This work is part of a pilot study on the genotypic characterisation of SCD patients in Ibadan, Nigeria carried out between September 2016 and March, 2018. The same number of apparently healthy individuals without SCD from the community were also recruited. Participants were aged 3 years – 69 years. Venous blood was collected from each participant and 3 mL was added to ethylenediaminetetraacetic acid (EDTA) bottles for complete blood count, HPLC, and DNA extraction at the Genetics and Bioethics Research Unit laboratory, College of Medicine, University of Ibadan, Nigeria. DNA amplification, sequencing and variant analysis were carried out at the Haematology-Oncology Laboratory, Department of Medicine, University of Chicago. More details on the demographic and clinical parameters, sampling, laboratory analyses, and DNA extraction from whole blood have been previously described.^18^ Samples from a subset of 26 individuals (from another health facility, Ring-Road State Hospital, Ibadan) with levels of HbA_2_ > 3% and/or HbF > 10% were genotyped along with the recruited participants because the haematological profiles were suggestive of β-thalassaemia trait.

### Sample size determination

A total of 130 participants were recruited in each group (*n* = 260). The required sample size was estimated using the formula for comparing two proportions, assuming the prevalence of β-thalassaemia trait to be 6% among individuals with SCD and 20% among those without SCD. With a two-sided significance level (α) = 0.05 and 90% statistical power, the corresponding effect size (absolute difference) was 0.14. This calculation yielded a minimum of 116 participants per group, which was increased to 130 per group to accommodate potential non-response or data loss. At these parameters, the 95% confidence level and an estimated margin of error of ± 0.08 around the expected difference ensured adequate precision for detecting a statistically significant difference in β-thalassaemia trait prevalence between groups.

### Exclusion criteria

Individuals with SCD who had undergone blood transfusion within three months of the study recruitment were excluded from enrolment.

### Polymerase chain reaction amplification of the β-globin gene

The human β-globin gene is located on chromosome 11p15.4, GRCh38:5225464–5227071. It is 1608 base pairs (bp) long and contains three exons and two introns.^21^ This region was amplified in three separate polymerase chain reaction (PCR) reactions using three different sets of primers (Supplementary Table 1). The regions amplified by each set of primers are shown in Supplementary Figure 1. Each 15 µL PCR reaction contained 200 µM of each dNTP, 1.5 µL of 10x PCR buffer, 2.5 mM MgCl_2,_ 0.5 U Taq polymerase, 1.5 µL of 20 ng/µL of DNA template, and 10 µM of forward and reverse primers. PCR reactions were conducted in a thermocycler (PTC-200 Peltier Thermal Cycler; MJ Research Inc., Waltham, Massachusetts, United States) with an initial denaturation step at 98 °C for 2 min, followed by 35 cycles of 98 °C denaturation for 45 s, 63 °C annealing temperature for 45 s, and 72 °C extension for 1 min 30 s. A final 10-min extension at 72 °C was used to complete the reaction. A 5 µL aliquot of each amplicon was analysed by electrophoresis on a 1.5% agarose gel in tris-acetate EDTA buffer at 120 volts for 45 min. DNA samples from the 26 SCD patients from another study cohort were sequenced along with samples in this study. Only the first and third set of primers were used on this set of samples.

**FIGURE 1:**
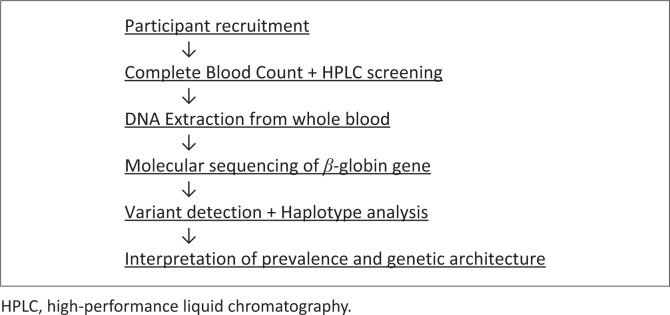
Diagnostic algorithm used to identify β-thalassaemia trait and analyse β-globin gene variants in the study population.

**TABLE 1:** Single nucleotide polymorphisms and mutations seen within the β-globin gene, their frequencies and significance.

SNP rs number	HGVS nomenclature	Allele frequency in cases†	Allele frequency in controls†	Allele frequency in YRI‡	Allele frequency in Africans‡	Global Allele frequency‡	ClinVar annotation
rs713040	NC_000011. 10:g.5227013A>G	1.000	0.892	0.894	0.884	0.7140	Synonymous mutation
rs33930165	NC_000011. 10:g.5227003C>T	0.058	0.018 (*n* = 4, heterozygous)	0.028	0.013	0.0030	Missense variant HbC, pathogenic
rs334	NC_000011. 10:g.5227002T>A	0.942	0.085 (*n* = 20, heterozygous)	0.139	0.100	0.0270	Missense variant HbS, pathogenic
rs33915217	NC_000011. 10:g.5226925C>A	-	-	0.000	0.000	0.0000	β^+^-thal, β-thal major
rs111851677	NC_000011. 10:g.5226822A>G	-	0.014 (*n* = 3, heterozygous)	0.019	0.022	0.0060	β^+^-thal, benign (conflicting submission)
rs10768683	NC_000011. 10:g.5226561C>G	1.000	0.892	0.894	0.890	0.7200	Intron variant, benign
rs7480526	NC_000011. 10:g.5226503A>C	-	0.256	0.338	0.379	0.3690	Intron variant, benign
rs7946748	NC_000011. 10:g.5226496G>A	-	0.119	0.065	0.065	0.0990	Intron variant, benign
rs113152027	NC_000011. 10:g.5226357G>A	-	0.026	0.009	0.007	0.0020	Intron variant, benign
rs1609812	NC_000011. 10:g.5225911G>A	1.000	0.889	0.894	0.884	0.7140	Intron variant, benign
rs537944366	NC_000011. 10:g.5225542T>C	-	0.0043 (*n* = 1, heterozygous)	-	0.002	0.0004	3’ UTR variant, uncertain significance

SNP, single nucleotide polymorphism; HGVS, Human Genome Variation Society; YRI, Yoruba in Ibadan; UTR, untranslated region; β^+^-thal, β-thalassaemia trait.

† , 2*n* = 246;

‡ , 1000 Genomes Phase 3 Project.

### Cleaning of β-globin gene amplicons for sequencing

Amplicons were cleaned prior to sequencing. Each 10 µL reaction contained 1 µL Shrimp Alkaline Phosphatase Buffer (10x), 0.5 µL of 1 U/µL Shrimp Alkaline Phosphatase, 0.1 µL of 20 U/µL Exo 1, 3.4 µL of PCR grade water and 5 µL of amplicons. This was then incubated at 37 °C for 45 mins, 95 °C for 15 mins and 4 °C indefinitely. A 4 µL aliquot of the cleaned amplicons was submitted for Sanger sequencing.

### Analysis of amplicon sequences

The sequences of the 1.6 kb β-globin gene amplicons were read and single nucleotide polymorphisms (SNPs) were detected using the Sequencher software version 4.2 (Gene Codes Corporation, Ann Arbor, Michigan, United States). The reference sequence of the GRCh38·p14 assembly with accession number NG_059281.1 was used as a template for sequence comparisons. The HbVar database^2^ was used to determine the rs numbers of the SNPs found within the amplicons, and dbSNP,^22^ ClinVar,^23^ Ensembl Variation database^24^ were used to determine the significance of each of the SNPs and allele frequencies in Africa and across the globe.

### Haplotype analysis

Using Haploview version 4.2 (Broad Institute, Cambridge, Massachusetts, United States),^25^ the SNPs detected in individuals without SCD were analysed in one haplotype block since the full length of the amplicon is less than 2 kb. Haploview was used to determine haplotype frequencies and SNPs in linkage disequilibrium within the β-globin gene. The diagnostic testing strategy employed in this study is summarised in Figure 1.

### Statistical analysis

Allele frequencies of SNPs detected in this study were compared with those found in the Ensembl 1000 Genomes Phase 3 project using Student’s *t*-test.

## Results

### Polymerase chain reaction amplicons of the β-globin gene

Three different sets of amplicons of size 774 bp, 645 bp and 574 bp were obtained from the PCR reactions (Supplementary Figure 2).

**FIGURE 2:**
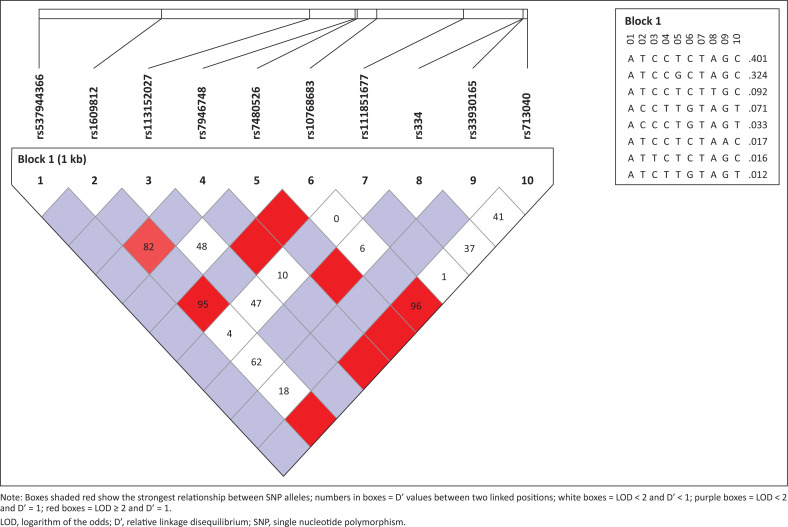
Haplotypes found within the β-globin gene, their frequencies and relationships generated by Haploview 4.2.

### Single nucleotide polymorphisms in the β-globin gene

Eleven variants (SNPs and mutations) were found in the study population (Table 1). Ten SNPs were found in the non-SCD group. These include rs713040, rs33930165, rs334, rs111851677, rs10768683, rs7480526, rs7946748, rs1609812, rs537944366, and rs113152027. Some of the SNPs in the β-globin gene are in linkage disequilibrium. Worthy of note are the relationships between rs1609812, rs7946748, rs10768683 and rs713040, with relative linkage disequilibrium (D’) of 0.824 – 1.0 and *r*^2^ of 0.508 – 0.887 for each pair of the SNPs (Table 2), three of which were the only SNPs (rs713040, rs10768683 and rs1609812) found in each SCD patient. The 11th variant – rs33915217 was found in one patient in the separate SCD cohort.

**TABLE 2:** Relative linkage disequilibrium and *r*-square values between the observed single nucleotide polymorphisms in the β-globin gene as generated by Haploview 4.2.

rs numbers	rs numbers								
	rs537944366	rs1609812	rs113152027	rs7946748	rs7480526	rs10768683	rs111851677	rs334	rs33930165
***D*’ values**									
rs1609812	1.000	-	-	-	-	-	-	-	-
rs113152027	1.000	1.000	-	-	-	-	-	-	-
rs7946748	1.000	0.824	1.000	-	-	-	-	-	-
rs7480526	1.000	1.000	0.480	1.000	-	-	-	-	-
rs10768683	1.000	0.955	1.000	1.000	1.000	-	-	-	-
rs111851677	1.000	0.043	0.478	0.102	1.000	0.000	-	-	-
rs334	0.050	0.620	0.280	0.360	1.000	0.060	1.000	-	-
rs33930165	1.000	0.180	1.000	1.000	1.000	1.000	1.000	1.000	-
rs713040	1.000	1.000	1.000	1.000	1.000	0.960	0.011	0.379	0.412
***r*^2^ values**									
rs1609812	0.001	-	-	-	-	-	-	-	-
rs113152027	0.000	0.003	-	-	-	-	-	-	-
rs7946748	0.000	0.508	0.002	-	-	-	-	-	-
rs7480526	0.008	0.064	0.011	0.048	-	-	-	-	-
rs10768683	0.001	0.776	0.004	0.636	0.076	-	-	-	-
rs111851677	0.000	0.000	0.151	0.002	0.032	0.000	-	-	-
rs334	0.040	0.040	0.050	0.070	0.370	0.000	0.050	-	-
rs33930165	0.000	0.000	0.001	0.002	0.011	0.003	0.000	0.050	
rs713040	0.000	1.000	0.004	0.676	0.073	0.887	0.000	0.002	0.001

*D*’, relative linkage disequilibrium.

### Prevalence of β-thalassaemia in individuals with and without sickle cell disease

For this genetic analysis, participants with incomplete or inconclusive haematology or sequencing results were excluded. Results from 123 SCD patients, 117 non-SCD participants and the separate cohort of 26 SCD patients (*n* = 266) were analysed. No β-thalassaemia mutation was found in the SCD patients in the study main group, while a mutation that has been associated with benign β-thalassaemia trait (rs111851677) was found in three individuals of the non-SCD group in the heterozygous state, at a frequency of 0.014. These three individuals had normal haematocrit > 36%, HbF levels < 1%, and HbA_2_ levels < 3%. One of the individuals had a low mean corpuscular volume of 76.8% and an mean corpuscular haemoglobin of 23.8. One of the SCD patients in the separate cohort was found to have a moderately severe β-thalassaemia intermedia mutation (rs33915217C > A) (Supplementary Figure 3). This SNP is an intronic variant at chr11:5,226,925 (hg38). The patient had HbA at 8%, HbA_2_ at 4.8%, HbF at 11.9% and mean corpuscular volume at 67.9%. The full haematological parameters are also shown in Supplementary Figure 3.

### Single nucleotide polymorphism haplotypes and their relationship

Eight haplotypes were discovered in the entire study population, including both SCD and non-SCD individuals. The haplotypes were grouped into two distinct ancestral haplotypes (Figure 2). The major ancestral haplotypes occurred at a total frequency of about 0.850 and the minor ancestral haplotypes occurred at a total frequency of 0.116 (Table 3). Four SNPs (rs713040, rs10768683, rs7946748 and rs1609812) differentiate the major and minor ancestral haplotypes, and they are in linkage disequilibrium (Table 2). Only one haplotype (TAGGAGAACG), a variant of the major ancestral haplotype, was found in all the participants with SCD in the homozygous state (HbSS) and another variant (TAGGAGATTG) was associated with haemoglobin C (HbC) (found in those with HbSC and HbAC genotype). The minor ancestral haplotype and its variants were found in 27 (24%) non-SCD participants in the heterozygous state; no participant had the homozygous state.

**TABLE 3:** Partitioning of haplotypes into major and minor ancestral haplotypes, and single nucleotide polymorphism allele differences between the haplotypes.

	Major ancestral haplotype	Frequency	Minor ancestral haplotype	Frequency
1	TAGGAGATCG	0.401	TGGAACATCA	0.071
2	TAGGCGATCG	0.324	TGGGACATCA	0.033
3	TAGGAGAACG†	0.092	TAGAACATCA	0.012
4	TAGGAGATTG‡	0.017	-	-
5	TAAGAGATCG	0.016	-	-
**Total frequency**		**0.850**	-	**0.116**

Note: Green highlight shows differing single nucleotide polymorphism (SNP) alleles between the major and minor haplotypes, and yellow highlight shows differing SNP alleles between the ancestral haplotypes and their variants.

† , Haplotype found in HbS carriers (pathogenic mutation rs334 at position 8);

‡ , Haplotype found in HbC carriers (pathogenic mutation rs33930165 at position 9).

## Discussion

The β-globin gene is highly polymorphic, harbouring numerous mutations that give rise to various haemoglobinopathies. These disorders are often population-specific, with unique combinations of mutations occurring in different regions of the world.^7,26^ β-thalassaemia, for instance, is caused by over 300 known mutations in the β-globin gene.^2,27^ In our genotyping study of 123 SCD patients in the main cohort, no known β-thalassaemia mutations were detected. Only one individual, an SCD patient in the separate sub-cohort, was found to carry a pathogenic β^+^ thalassaemia mutation (rs33915217C > A), which is typically associated with β-thalassaemia intermedia in the homozygous or compound heterozygous state. This sub-cohort was analysed because of elevated HbA_2_ and/or HbF levels, which in clinical practice often lead to presumptive diagnosis of β-thalassaemia trait based on HPLC alone.

The rs33915217 SNP has three rare alleles: C > A, C > G, and C > T^2^. All three disrupt normal splicing at the 5′ splice site, impairing mRNA processing and resulting in reduced or absent β-globin production.^2,28^ The A allele, found in our study, is particularly rare, and has previously been reported only in two individuals from Europe—one heterozygous and one homozygous.^28^ To our knowledge, this is the first report of this allele in both an African individual and an SCD patient. It was absent from the 1000 Genomes Phase 3 data set but is recorded at extremely low frequency (0.000009)^24^ in the gnomAD exome (non-Finnish European) data set.

The rs111851677 variant (IVS-I-108), found in three individuals without SCD in the heterozygous state, has been previously reported in β-thalassaemia carriers from Cuba,^29^ Iran,^30^ and Greece.^31^ Though its clinical impact is debated, it may impair RNA splicing because of its location near the 3′ splice site.^29,32^ In our study, however, individuals carrying this variant showed normal haematological profiles, suggesting a benign role.

Our findings support earlier studies from Nigeria^13,16^ that reported β-thalassaemia mutations to be rare (< 1%) in the population. This contrasts with recent reports suggesting a prevalence of 6% to 10% in SCD patients and 20% to 25% in the general Nigerian population.^9,10,14,15^ The discrepancy likely stems from methodological differences, as these studies relied solely on haematologic indices and HPLC thresholds (HbA_2_ > 3.0% – 3.5%, HbF > 1%) to infer β-thalassaemia trait without molecular confirmation. Such screening-based approaches are prone to overestimation because of conditions that elevate HbA_2_, including α-thalassaemia,^16,18^ vitamin B_12_ or folate deficiency, antiretroviral therapy, and thyroid disorders.^19^ In our study, all the individuals (except one) in both the main and separate cohorts with elevated HbA_2_ levels had no detectable β-thalassaemia mutations on genetic testing. Similar findings were reported by Inusa et al.,^16^ who found no underlying β-thalassaemia mutations despite elevated HbA_2_ levels (up to 7.4%). This underscores the need for standardised diagnostic methods integrating molecular genotyping.

Given the high prevalence of α thalassaemia in Nigeria (estimated at 35% – 45%),^18,20^ this could contribute significantly to spurious elevations in HbA_2_ and possible misdiagnosis of β-thalassaemia trait. Interestingly, since β-thalassaemia trait can ameliorate SCD symptoms, its frequency would be expected to be higher in SCD patients than in the general population. However, recent HPLC-based studies report the reverse,^9,10^ further emphasising the limitations of relying solely on haematologic indices for diagnosis.

Apart from rs33915217 (β-thalassaemia), rs334 (HbS), and rs33930165 (HbC), all other variants identified in our study were non-pathogenic intronic SNPs. Notably, rs713040G—which has a higher global frequency than its reference allele—was found in 100% of SCD patients, compared to ~80% in non-SCD, African, and global populations. Similarly, rs33930165 (HbC mutation) was observed in 1.8% of the non-SCD cohort, aligning with its expected prevalence in African populations (0.013–0.028).^24^ The rs334 (HbS mutation) had a frequency of 0.085 in the non-SCD group, which is consistent with African averages and lower than in the Yoruba in Ibadan reference population.

Other intronic variants found in this study (rs10768683, rs7480526, rs7946748, rs113152027, rs1609812) displayed population frequencies consistent with African ancestry. One rare 3′ untranslated region variant, rs537944366, was detected in only one individual. This variant, absent in the Yoruba in Ibadan and only recorded in Mende in Sierra Leone and gnomAD non-Finnish Europeans, is being reported for the first time in a Nigerian (Yoruba) population.

Our haplotype analysis revealed two ancestral haplotypes: a major haplotype (frequency: 0.850), typical of African populations, and a minor haplotype (frequency: 0.116), atypical for Africans and more common in South and East Asian populations. These haplotypes are differentiated by four SNPs in linkage disequilibrium (rs713040, rs10768683, rs7946748, rs1609812). The minor ancestral haplotype and its variants were found in 27 (24%) non-SCD participants in the heterozygous state; no participant had the homozygous state. Hardy-Weinberg expectations suggest a frequency too low to detect given the current sample size.

Only the major haplotype (TAGGAGAACG) was associated with HbS and HbC mutations. Interestingly, the SCD patient with the β-thalassaemia intermedia mutation was heterozygous for both haplotypes, linking the pathogenic variant to the minor ancestral haplotype. The rs33915217 β-thalassaemia mutation was found on the minor haplotype, supporting the idea of genetic admixture or historical gene flow into this population. This pattern suggests that HbS and HbC mutations in this population have likely evolved on a common African haplotypic background and have undergone minimal recombination.

Our findings challenge the assumption of a high prevalence of β-thalassaemia trait in Nigeria. The findings also validate the robustness of the sequencing and analysis pipeline. Seven SNPs from our data showed the same linkage disequilibrium pattern in Yoruba in Ibadan individuals from the 1000 Genomes Project,^33^ reinforcing the accuracy of our haplotype deductions.

### Limitations

The study population is from a specific region of the country that is predominantly of Yoruba ethnicity, which may not represent the full genetic diversity of the Nigerian population. The sample size, although statistically adequate for detecting group differences, may not have been large enough to detect very rare β-thalassaemia variants. Only the β-globin gene region was sequenced; hence, mutations in regulatory or flanking regions may have been missed.

### Conclusion

This study demonstrates that β-thalassaemia trait could be rare in both SCD and non-SCD individuals in the Yoruba population of Nigeria, and is associated with a minor ancestral haplotype likely introduced through genetic admixture. Haematologic parameters such as elevated HbA_2_ and HbF, while being useful screening tools, are insufficient for the definitive diagnosis of β-thalassaemia trait in African populations because of confounding factors such as α-thalassaemia, infections, and folate deficiency. Genetic confirmation is essential to avoid misdiagnosis, especially in regions with high prevalence of SCD, where correct genotyping is crucial for accurate disease classification, appropriate clinical management, and genetic counseling.

## References

[1] Cao A, Galanello R, Rosatelli MC. Genotype-phenotype correlations in beta-thalassaemias. Blood Rev. 1994;8(1):1–12. 10.1016/0268-960X(94)90002-7 8205005

[2] A database of human haemoglobin variants and thalassaemias [homepage on the Internet]. 2024 [cited 2024 Jun 17]. Available from: https://globin.bx.psu.edu/cgi-bin/hbvar/query_vars3

[3] Cao A, Moi P. Regulation of the globin genes. Pediatr Res. 2002;51(4):415–421. 10.1203/00006450-200204000-00003 11919323

[4] Weatherall DJ. Phenotype-genotype relationships in monogenic disease: Lessons from the thalassaemias. Nat Rev Genet. 2001;2(4):245–255. 10.1038/35066048 11283697

[5] Galanello R, Origa R. Beta-thalassaemia. Orphanet J Rare Dis. 2010;5:11. 10.1186/1750-1172-5-11 20492708 PMC2893117

[6] Kattamis A, Forni GL, Aydinok Y, Viprakasit V. Changing patterns in the epidemiology of β-thalassaemia. Eur J Haematol. 2020;105(6):692–703. 10.1111/ejh.13512 32886826 PMC7692954

[7] Flint J, Harding RM, Boyce AJ, Clegg JB. The population genetics of the haemoglobinopathies. Baillieres Clin Haematol. 1998;11(1):1–51. 10.1016/S0950-3536(98)80069-3 10872472

[8] Kotila T. Beta thalassaemia in Nigeria: Myth or fact? Afr J Med Med Sci. 2013;42(3):261–264. 24579388

[9] Kotila TR, Adeyemo AA, Mewoyeka OO, Shokunb WA. Beta thalassaemia trait in western Nigeria. African Health Sci. 2009;9(1):46–48. PMC2932515 20842242

[10] Isaiah A, Kalle Kwaifa I, Abdulrahman Y, Audu Sunday O. Prevalence of thalassaemia in Nigeria: Pathophysiology and clinical manifestations. Clin Med Health Res J. 2024;4(2):806–815. 10.18535/cmhrj.v4i2.325

[11] Willcox M, Björkman A, Brohult J. Falciparum malaria and beta-thalassaemia trait in northern Liberia. Ann Trop Med Parasitol. 1983;77(4):335–347. 10.1080/00034983.1983.11811722 6357119

[12] Rao E, Chandraker SK, Singh MM, Kumar R. Global distribution of β-thalassaemia mutations: An update. Gene. 2024;896:148022. 10.1016/j.gene.2023.148022 38007159

[13] Esan GJ. The thalassaemia syndromes in Nigeria. Br J Haematol. 1970;19(1):47–56. 10.1111/j.1365-2141.1970.tb01600.x 5453916

[14] Adeyemo T, Ojewunmi O, Oyetunji A. Evaluation of high performance liquid chromatography (HPLC) pattern and prevalence of beta-thalassaemia trait among sickle cell disease patients in Lagos, Nigeria. Pan Afr Med J. 2014;18:71. 10.11604/pamj.2014.18.71.4239 25400838 PMC4230225

[15] Vincent O, Oluwaseyi B, James B, Saidat L. Coinheritance of B-thalassaemia and sickle cell anaemia in Southwestern Nigeria. Ethiop J Health Sci. 2016;26(6):517–522. 10.4314/ejhs.v26i6.3 28450766 PMC5389070

[16] Inusa BP, Daniel Y, Lawson JO, Dada J, Matthews CE, et al. Sickle cell disease screening in Northern Nigeria: the coexistence of β-thalassemia inheritance. Pediatr Ther. 2015;5:262. 10.4172/2161-0665.1000262

[17] Platt OS, Thorington BD, Brambilla DJ, et al. Pain in sickle cell disease. Rates and risk factors. N Engl J Med. 1991;325(1):11–16. 10.1056/NEJM199107043250103 1710777

[18] Fasola FA, Babalola OA, Brown BJ, Odetunde A, Falusi AG, Olopade O. The effect of alpha thalassaemia, HbF and HbC on haematological parameters of sickle cell disease patients in Ibadan, Nigeria. Mediterranean J Hematol Infect Dis. 2022;14(1):e2022001. 10.4084/MJHID.2022.001 PMC8747010 35070208

[19] Needs T, Gonzalez-Mosquera LF, Lynch DT. Beta thalassemia. In: StatPearls [Internet]. Treasure Island (FL): StatPearls Publishing; 2025 Jan– [cited 2023 May 01]. Available from: https://www.ncbi.nlm.nih.gov/books/NBK531481/30285376

[20] Falusi AG, Esan GJ, Ayyub H, Higgs DR. Alpha-thalassaemia in Nigeria: Its interaction with sickle-cell disease. Eur J Haematol. 1987;38(4):370–375. 10.1111/j.1600-0609.1987.tb00013.x 3609256

[21] HBB haemoglobin subunit beta [*Homo sapiens* (Human)] [homepage on the Internet]. 2024 [cited 2024 May 20]. Available from: https://www.ncbi.nlm.nih.gov/gene/3043

[22] dbSNP short genetic variations [homepage on the Internet]. 2024 [cited 2024 Jul 09]. Available from: https://www.ncbi.nlm.nih.gov/snp/rs33915217#clinical_significance

[23] ClinVar [homepage on the Internet]. 2024 [cited 2024 Jun 24]. Available from: https://www.bing.com/search?q=ClinVar+(https%3A%2F%2Fwww.ncbi.nlm.nih.gov%2Fclinvar%2F)&cvid=c0eab5d3df024dd4885f4c1eac1e9a73&gs_lcrp=EgRlZGdlKgYIABBFGDsyBggAEEUYO9IBBzUxNWowajmoAgiwAgE&FORM=ANAB01&PC=HCTS

[24] Variation [homepage on the Internet]. 2024 [cited 2024 Jul 09]. Available from: http://www.ensembl.org/info/genome/variation/index.html

[25] Barrett JC, Fry B, Maller J, Daly MJ. Haploview: Analysis and visualization of LD and haplotype maps. Bioinformatics. 2005;21(2):263–265. 10.1093/bioinformatics/bth457 15297300

[26] Henderson SJ, Timbs AT, McCarthy J, et al. Ten years of routine α- and β-globin gene sequencing in UK haemoglobinopathy referrals reveals 60 novel nutations. Haemoglobin. 2016;40(2):75–84. 10.3109/03630269.2015.1113990 26635043

[27] Thein SL. The molecular basis of β-thalassaemia. Cold Spring Harb Perspect Med. 2013;3(5):a011700. 10.1101/cshperspect.a011700 23637309 PMC3633182

[28] Atweh GF, Wong C, Reed R, et al. A new mutation in IVS-1 of the human beta globin gene causing beta thalassaemia due to abnormal splicing. Blood. 1987;70(1):147–151. 10.1182/blood.V70.1.147.147 2439149

[29] Muñiz A, Martinez G, Lavinha J, Pacheco P. Beta-thalassaemia in Cubans: Novel allele increases the genetic diversity at the HBB locus in the Caribbean. Am J Hematol. 2000;64(1):7–14. 10.1002/(SICI)1096-8652(200005)64:1<7::AID-AJH2>3.3.CO;2-M 10815781

[30] Badens C, Jassim N, Martini N, Mattei JF, Elion J, Lena-Russo D. Characterization of a new polymorphism, IVS-I-108 (T-->C), and a new beta-thalassaemia mutation, -27 (A-->T), discovered in the course of a prenatal diagnosis. Haemoglobin. 1999;23(4):339–344. 10.3109/03630269909090749 10569722

[31] Boussiou M, Karababa P, Sinopoulou K, Tsaftaridis P, Plata E, Loutradi-Anagnostou A. The molecular heterogeneity of beta-thalassaemia in Greece. Blood Cells Mol Dis. 2008;40(3):317–319. 10.1016/j.bcmd.2007.11.003 18096416

[32] Vinciguerra M, Cassarà F, Cannata M, et al. Phenotypic evaluations of HBB:c.93-23T&gt;C, a nucleotide substitution in the IVS I nt 108 of β-globin gene. J Clin Pathol. 2018;71(4):298–302. 10.1136/jclinpath-2017-204651 28794124

[33] Linkage disequilibrium data [homepage on the Internet]. 2024 [cited 2024 Jun 18]. Available from: http://www.ensembl.org/Homo_sapiens/Location/LD/ajax?db=core;g=ENSG00000244734;r=11%3A5225464-5227071;t=ENST00000647020;pop1=373538

